# Calibration of patient-specific boundary conditions for coupled CFD models of the aorta derived from 4D Flow-MRI

**DOI:** 10.3389/fbioe.2023.1178483

**Published:** 2023-05-11

**Authors:** Scott MacDonald Black, Craig Maclean, Pauline Hall Barrientos, Konstantinos Ritos, Alistair McQueen, Asimina Kazakidi

**Affiliations:** ^1^ Department of Biomedical Engineering, University of Strathclyde, Glasgow, United Kingdom; ^2^ Research and Development, Terumo Aortic, Glasgow, United Kingdom; ^3^ Clinical Physics, Queen Elizabeth University Hospital, NHS Greater Glasgow and Clyde, Glasgow, United Kingdom; ^4^ Department of Mechanical and Aerospace Engineering, University of Strathclyde, Glasgow, United Kingdom; ^5^ Department of Mechanical Engineering, University of Thessaly, Volos, Greece; ^6^ Department of Biomedical Engineering, University of Glasgow, Glasgow, United Kingdom

**Keywords:** CFD, 4D Flow-MRI, boundary conditions, calibration, Windkessel, aortic dissection, patient-specific

## Abstract

**Introduction:** Patient-specific computational fluid dynamics (CFD) models permit analysis of complex intra-aortic hemodynamics in patients with aortic dissection (AD), where vessel morphology and disease severity are highly individualized. The simulated blood flow regime within these models is sensitive to the prescribed boundary conditions (BCs), so accurate BC selection is fundamental to achieve clinically relevant results.

**Methods:** This study presents a novel reduced-order computational framework for the iterative flow-based calibration of 3-Element Windkessel Model (3EWM) parameters to generate patient-specific BCs. These parameters were calibrated using time-resolved flow information derived from retrospective four-dimensional flow magnetic resonance imaging (4D Flow-MRI). For a healthy and dissected case, blood flow was then investigated numerically in a fully coupled zero dimensional-three dimensional (0D-3D) numerical framework, where the vessel geometries were reconstructed from medical images. Calibration of the 3EWM parameters was automated and required ~3.5 min per branch.

**Results:** With prescription of the calibrated BCs, the computed near-wall hemodynamics (time-averaged wall shear stress, oscillatory shear index) and perfusion distribution were consistent with clinical measurements and previous literature, yielding physiologically relevant results. BC calibration was particularly important in the AD case, where the complex flow regime was captured only after BC calibration.

**Discussion:** This calibration methodology can therefore be applied in clinical cases where branch flow rates are known, for example, via 4D Flow-MRI or ultrasound, to generate patient-specific BCs for CFD models. It is then possible to elucidate, on a case-by-case basis, the highly individualized hemodynamics which occur due to geometric variations in aortic pathology high spatiotemporal resolution through CFD.

## 1 Introduction

The aorta is the largest arterial segment of the human systemic circulation and exhibits a complex flow regime (Zamir et al., 1992; Fung et al., 2008; Konoura et al., 2013). This region can be affected by aortic dissection (AD), characterized by a primary intimal tear which results in the creation of a false lumen (FL), and additional secondary intraluminal tears (Tse et al., 2011; Liu et al., 2018; Xu et al., 2018). This FL forms when blood flows through the intimal tear and into the medial layer of the aortic wall, creating a secondary channel which extends longitudinally beside the native lumen (Tse et al., 2011). As the FL demonstrates a proclivity to expand and potentially rupture, there is a risk of serious morbidity and mortality in the absence of intervention (Tse et al., 2011; Alimohammadi et al., 2014; Liu et al., 2018).

Capturing this complex blood flow regime *in vivo* is challenging, but four-dimensional flow magnetic resonance imaging (4D Flow-MRI) presents a reliable, non-invasive tool for such analysis. Crucially, velocity is encoded in three principal spatial directions and time, permitting 3D evaluation of the dynamic evolution of blood flow throughout an entire cardiac cycle (Callaghan and Grieve, 2018; Alvarez et al., 2020). Fundamentally, this quantitative analysis can be performed *post hoc* at any point in a region of interest (ROI) due to complete volumetric coverage (Stankovic et al., 2014; Nayak et al., 2015). To date, 4D Flow-MRI has been used to observe and quantify a range of hemodynamic parameters including wall shear stress, peak velocity, flow rate and regurgitant fraction, in healthy and dissected aortae (Liu et al., 2018). Previous studies indicate however that the calculation of near-wall hemodynamic parameters like wall shear stress (WSS) via 4D Flow-MRI may be inaccurate due to poor spatial and temporal resolution (Callaghan and Grieve, 2018).

Computational fluid dynamics (CFD) models can overcome this limitation, portraying the distribution of near wall hemodynamics with unparalleled spatiotemporal resolution (Markl et al., 2016; Johnston et al., 2021a). Through CFD, it is also possible to investigate numerically the effect of isolated factors in a controlled environment, e.g., by setting different boundary conditions (BCs) to which the aortic flow regime is very sensitive (Kim et al., 2009; Romarowski et al., 2018). Utilizing CFD models to expand upon clinical data may aid clinicians with diagnostic decision making due to the ability to accurately replicate complex intra-aortic hemodynamics (Kim et al., 2009; Madhavan and Kemmerling, 2018). For example, these models may indicate sites of future dissection or aneurysm development (Tse et al., 2011).

Presently, it is not possible to model the entire systemic circulation in 3D due to lack of imaging resolution and the prohibitively expensive computational cost (Spilker and Taylor, 2010; Moghadam et al., 2013). Further, while distal vasculature accounts for most of the vascular resistance, the clinically relevant flow phenomena such as jet flow and recirculation in the case of AD, because of intraluminal tears, develop within larger vessels (Moghadam et al., 2013). Therefore, a multi-dimensional approach is required to incorporate all relevant domains in a unified model. As such, complex spatiotemporal flow behavior is solved in the high-fidelity 3D domain, while the effect of distal vasculature is estimated through computationally efficient, reduced order BCs (Kim et al., 2009; Boumpouli et al., 2020; Johnston et al., 2021b). To generate patient-specific CFD models, these BCs must be physiologically accurate, robust, and simple to implement on a parallel computing framework (Grinberg and Karniadakis, 2008).

A zero-dimensional (0D) 3-Element Windkessel Model (3EWM) is commonly employed at the outlet boundaries to describe the pressure-flow relationship due to distal vasculature (Pirola et al., 2017; Bonfanti et al., 2019; Hyde-Linaker et al., 2022). Clinical application of such BCs requires patient-specific tuning of the Windkessel parameters, which is not a trivial task, and for which there is no single, agreed upon methodology (Alimohammadi et al., 2014; Pirola et al., 2017). Previous studies describe root finding algorithms, Kalman filtering, and iterative calibration loops (Spilker and Taylor, 2010; Xiao, 2014). Often, these studies impose parameters which are calibrated based on invasive pressure measurements, empirical laws, and many require arterial pulse wave velocity (PWV) to be estimated (Kim et al., 2009; Pirola et al., 2017). These calibration approaches become extremely difficult in the presence of arterial pathology as highly individualized changes in vessel morphology create a chaotic flow regime which cannot be readily estimated (Alimohammadi et al., 2015; Fevola et al., 2021). Moreover, current calibration methodologies often rely on non-patient-specific data from previous literature, leading to further inaccuracies (Xu et al., 2018; Bonfanti et al., 2019).

4D Flow-MRI derived parameter calibration eradicates the need for assumptions since functional flow and anatomical information can be obtained in parallel from a single, non-invasive, non-ionising scan of the patient. Therefore, the aim of this study is to outline a methodology to generate patient-specific 3EWM BCs derived from retrospective 4D Flow-MRI images of a healthy and a dissected aorta. This study will then demonstrate the application of these BCs in the generation of a patient-specific, coupled 0D-3D CFD model for a healthy and dissected aorta as proof-of-concept examples. To the best of our knowledge, this has not previously been performed, and our results are the first to demonstrate the efficacy and value of such a framework.

## 2 Materials and methods

### 2.1 Data acquisition

**FIGURE 1:**
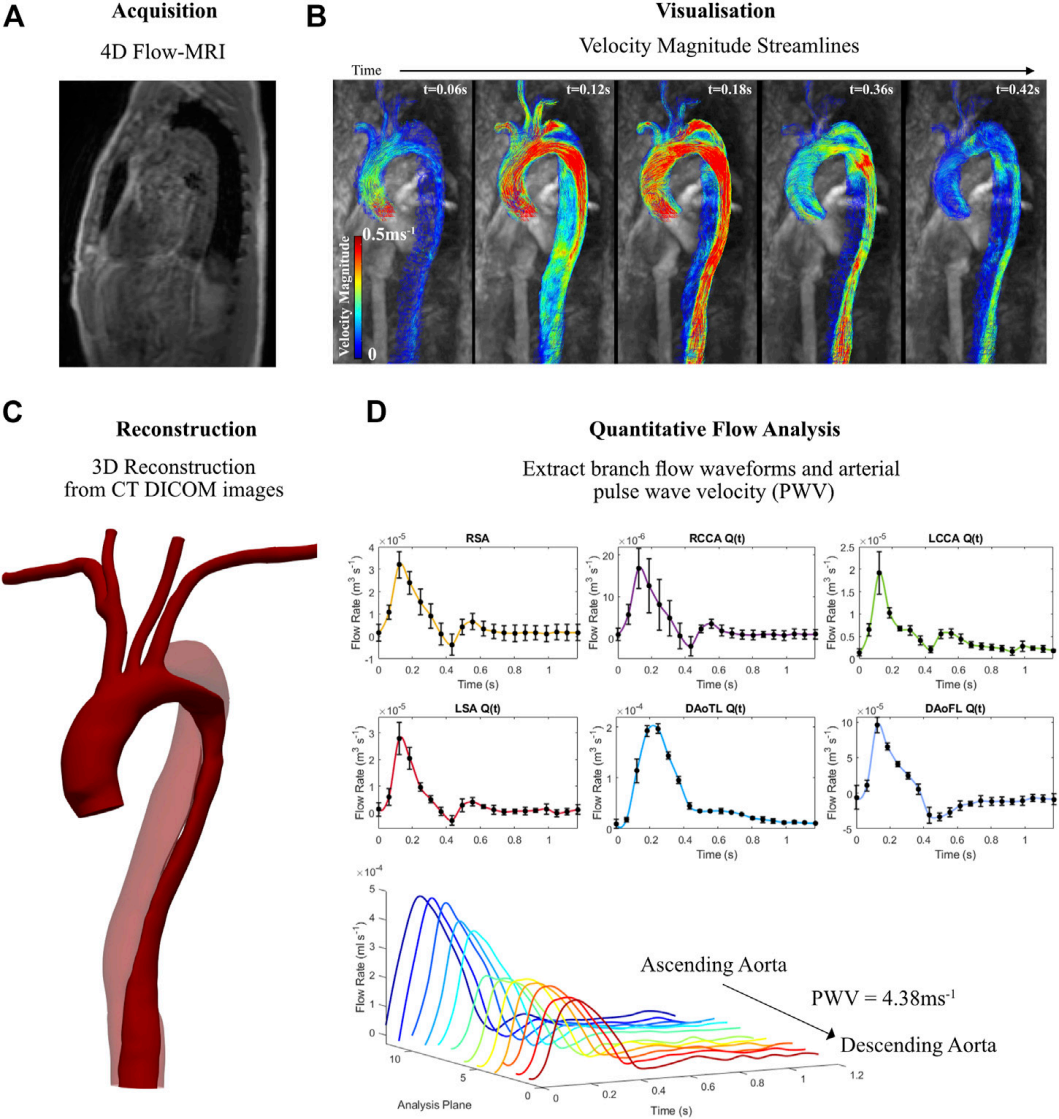
Information derived from 4D Flow-MRI and CT data for the aortic dissection patient, illustrating **(A)** 4D Flow-MRI acquisition, **(B)** visualisation of velocity streamlines on Circle Cardiovascular Imaging Software^®^ at multiple time points throughout the cardiac cycle (t = 0.06, 0.12, 0.18, 0.36, and 0.42 s), **(C)** reconstructed geometry of the dissected thoracic aorta, illustrating the true lumen (solid color) and the false lumen (transparent), and **(D)** branch flow waveforms and pulse wave velocity (PWV) extraction which were used to generate patient-specific 3-Element Windkessel boundary conditions.

Velocity encoding (VENC) 4D Flow-MRI images of the aortae of two patients, one healthy (33 year old male) and one with a dissected aorta (55 year old male), were acquired via a Siemens research 4D Flow-MRI sequence (WIP 785A). Figure 1 summarizes this methodology, showing the dissected aorta as an example. This sequence employed an acquired resolution of 3.6 × 2.4 × 2.6 mm^3^, an imaging volume of 80 × 160 × 60 mm^3^, and VENC of 1.5 ms^−1^ (Figures 1A, B). The repetition and echo time were 3.8 and 2.8 ms respectively, and 20 frames were acquired between each successive R-wave of the QRS signal of the electrocardiogram. The total scan time was ∼8 min. Computed tomography (CT) images were also obtained for the dissected aorta via helical contrast-enhanced CT angiography (CE-CTA) using 100 mL iodinated contrast media, in the absence of cardiac gating. Due to ethical implications of ionizing radiation, CT images were not obtained for the healthy volunteer.

### 2.2 3D arterial reconstruction

The thoracic aorta of the healthy volunteer was reconstructed from 4D Flow-MRI data using the methodology described in Black et al., (2023). In brief, a 3D magnetic resonance angiogram was created by deriving contrast from the instantaneous velocity magnitude of blood during systolic acceleration, peak systole, and systolic deceleration (Figures 1A, B). These images were then superimposed to create a temporal composite Digital Imaging and Communications in Medicine (DICOM) stack of images which exhibited high contrast in the vessel lumen.

For the AD case, multi-VENC MRI imaging is required to allow for the precise segmentation of the false lumen (Black et al., 2023). As this was not available, the geometry of the dissected aorta was reconstructed from CT images (Figure 1C). The image stack for each individual case was then processed in SimVascular^®^ (https://simvascular.github.io/), where semi-automatic segmentation was performed (Lan et al., 2018). 2D segmentations were created along the vessel centerlines via intensity thresholding, while manual corrections were applied to ensure true representation of the lumen. These contours were then lofted along the vessel centerlines and stitched together to generate a solid model. This was then smoothed with 10 iterations of decimation and constrained smoothing. Flow extensions were added at the inlet (5 × D) and outlets (10 × D), where D was the diameter of the terminal branch. These are extensions of the meshed domain at the inlets and outlets in the direction which is normal to the boundary face. Flow extensions improve numerical convergence, accuracy and stability of the CFD model by ensuring the location of the boundary faces do not influence results within the domain of interest (Kazakidi et al., 2009; Madhavan and Kemmerling, 2018).

### 2.3 CFD methodology

To discretize the 3D models for numerical investigation, a tetrahedral mesh was generated in Ansys ICEM CFD^®^. To resolve the viscous sublayer, an initial boundary layer height of 0.0015 m was prescribed to ensure y+ < 1 throughout the geometry. This y+ parameter is a non-dimensional measurement of distance from the first boundary prism layer to the mesh wall which determines the applicability of different methods for near-wall turbulence modelling. Thereafter, 11 additional prism layers were generated utilizing an exponential expansion ratio of 1.2 (i.e., $h_{n}=h_{1}e^{\left(n-1\right)p}$ , where $h_{1}$ is the initial height; n is the number of prism layers; $h_{n}$ is the height of each subsequent layer, and *p* is the exponent).

To ensure mesh independence, a grid convergence study was performed which evaluated time averaged wall shear stress (TAWSS) at multiple regions throughout the geometry where the most complex flow was expected. This included the area around each intraluminal tear, the supra-aortic branch ostia, and the innominate bifurcation. A total of 3.8 million elements (excluding flow extensions) were required to ensure mesh independence, such that the computed TAWSS was less than 2.5% different than the Richardson Extrapolation.

Blood flow was simulated by solving the 3D, time-dependent, incompressible, Reynolds-averaged Navier Stokes (RANS) equations for continuity and momentum corresponding to Eqs 1, 2, respectively (Alfonsi, 2009).

$$\frac{{\partial \bar{u}}_{i}}{{\partial x}_{i}}=0$$ (1)

$$\frac{{\partial \bar{u}}_{i}}{\partial t}+{\bar{u}}_{j}\frac{{\partial \bar{u}}_{i}}{{\partial x}_{j}}=-\frac{1}{\rho }\frac{\partial \bar{p}}{{\partial x}_{i}}+v\frac{{\partial^{2}\bar{u}}_{i}}{{\partial x}_{j}{\partial x}_{j}}-\frac{{\partial \tau }_{ij}}{{\partial x}_{j}},$$ (2)

where *i,j* = 1,2,3 (Einstein summation convention applies to repeated indices), u is the velocity of blood, ρ is the density of blood, v is the viscosity of blood, and p is the pressure. Eq. 2 separates velocity and pressure into mean ( $\bar{u}$ ; $\bar{p}$ *)* and fluctuating ( $u^{\prime }$ ; $p^{\prime }$ ) components, where $u=\bar{u}+u^{\prime }$ , $p=\bar{p}+p^{\prime }$ , and $\tau_{ij}=\bar{{u_{i}}^{\prime }{u_{j}}^{\prime }}$ (Alfonsi, 2009).

The governing Navier Stokes equations solved numerically in Ansys Fluent^®^ utilizing a finite volume method, a standard k-ω turbulence model, and the Pressure-Implicit with Splitting of Operators (PISO) algorithm at 10 iterations per time step (dt = 0.001s) (López et al., 2015). The standard k-ω turbulence model was utilized since it has been shown to be more accurate when compared to experimental data and maintain stability in regions of stagnation and high fluid acceleration which are common in AD cases (Song et al., 2003; Kabir et al., 2020). CFD Simulations were performed on a single node of the ARCHIE-WeSt cluster at the University of Strathclyde. These required ∼16 h on average to solve five cardiac cycles on 35 Intel Xeon Gold 6138 (Skylake) processors at 2.0 GHz and 4.8 GB RAM per core. Blood was assumed to be Newtonian due to high shear rates within the aorta, with a density of 1,060 kgm^−3^ and a dynamic viscosity, μ, of 0.004 Pa s (Boumpouli et al., 2021). Hemodynamic analysis was performed on the fifth cardiac cycle when time-periodicity was obtained, where pressure and flow rate altered by less than 1.5% in consecutive cardiac cycles. This was to ensure convergence for unsteady flows.

TAWSS and oscillatory shear index (OSI) were calculated via a user defined function (UDF) using the following definitions (Peiffer et al., 2013):

$$TAWSS=\frac{1}{T}\int_{0}^{T}\left|{\vec{\tau }}_{\omega }\right|dt$$ (3)

$$OSI=\frac{1}{2}\left(1-\frac{\left|\int_{0}^{T}{\vec{\tau }}_{\omega }dt\right|}{\int_{0}^{T}\left|{\vec{\tau }}_{\omega }\right|dt}\right)=\frac{1}{2}\left(1-\frac{\left|{\vec{\tau }}_{mean}\right|}{TAWSS}\right),$$ (4)

where ${\vec{\tau }}_{mean}=\frac{1}{T}\int_{0}^{T}{\vec{\tau }}_{\omega }dt$ , ${\vec{\tau }}_{\omega }$ is the instantaneous wall shear stress vector, dt is the time step, and T is the time for one full cardiac cycle. OSI characterizes the degree of shear reversal in pulsatile flow, ranging from 0 for unidirectional flow, to 0.5 which is indicative of a reversing flow with no mean direction of shear (Amaya et al., 2015).

### 2.4 Boundary conditions

**FIGURE 2:**
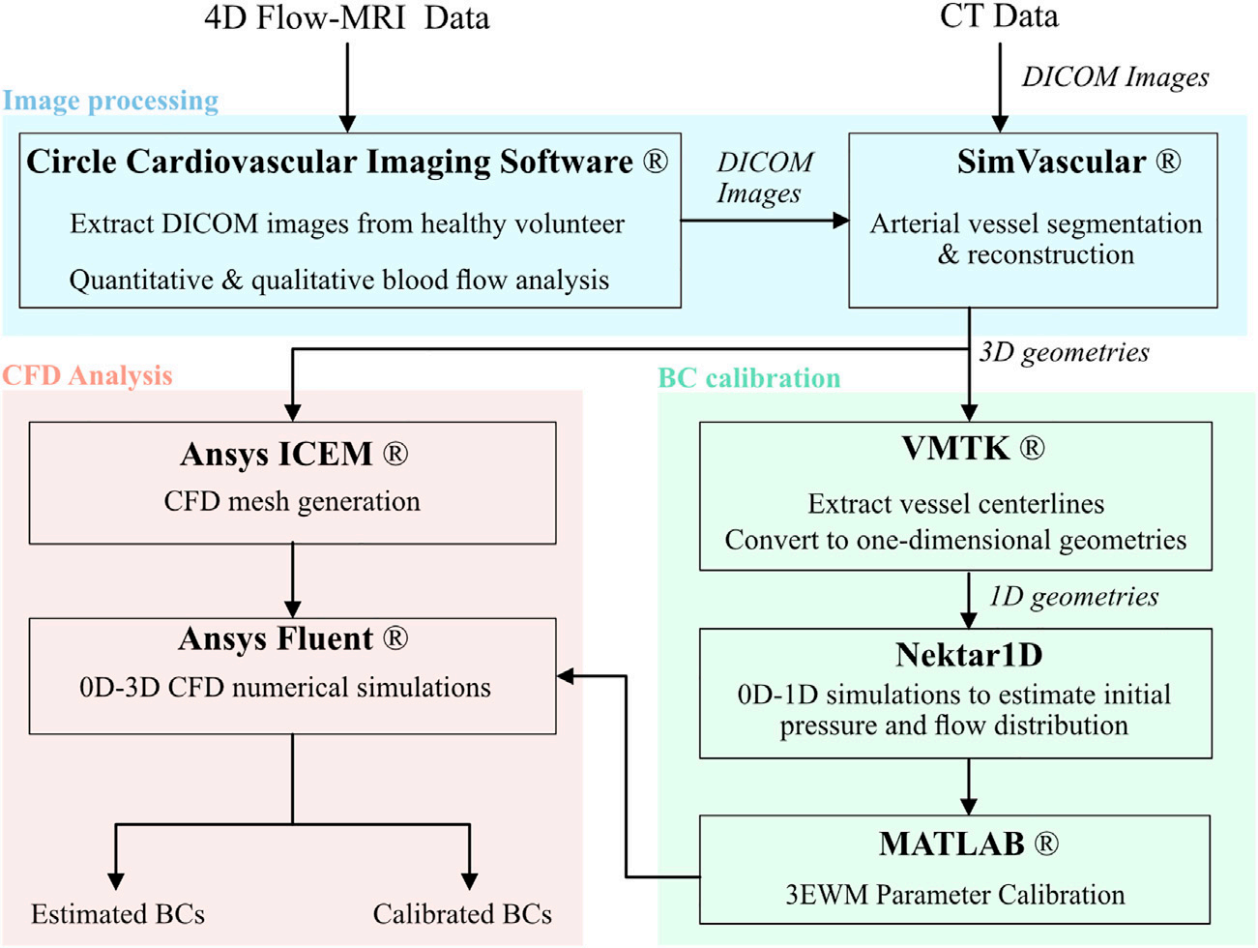
Flowchart of the methodology and software used to generate patient specific CFD models of the thoracic aortae including image processing, boundary condition calibration, and numerical analysis.

Outlet Windkessel BCs were estimated from geometric parameters and arterial PWV, while inlet waveforms were extracted directly from *in vivo* data. The outlet BCs were subsequently calibrated against 4D Flow-MRI-derived *in vivo* blood flow data at each branch of the thoracic aorta. Figure 2 details a flowchart of the calibration methodology and CFD analysis.

#### 2.4.1 Inlet profiles

**FIGURE 3:**
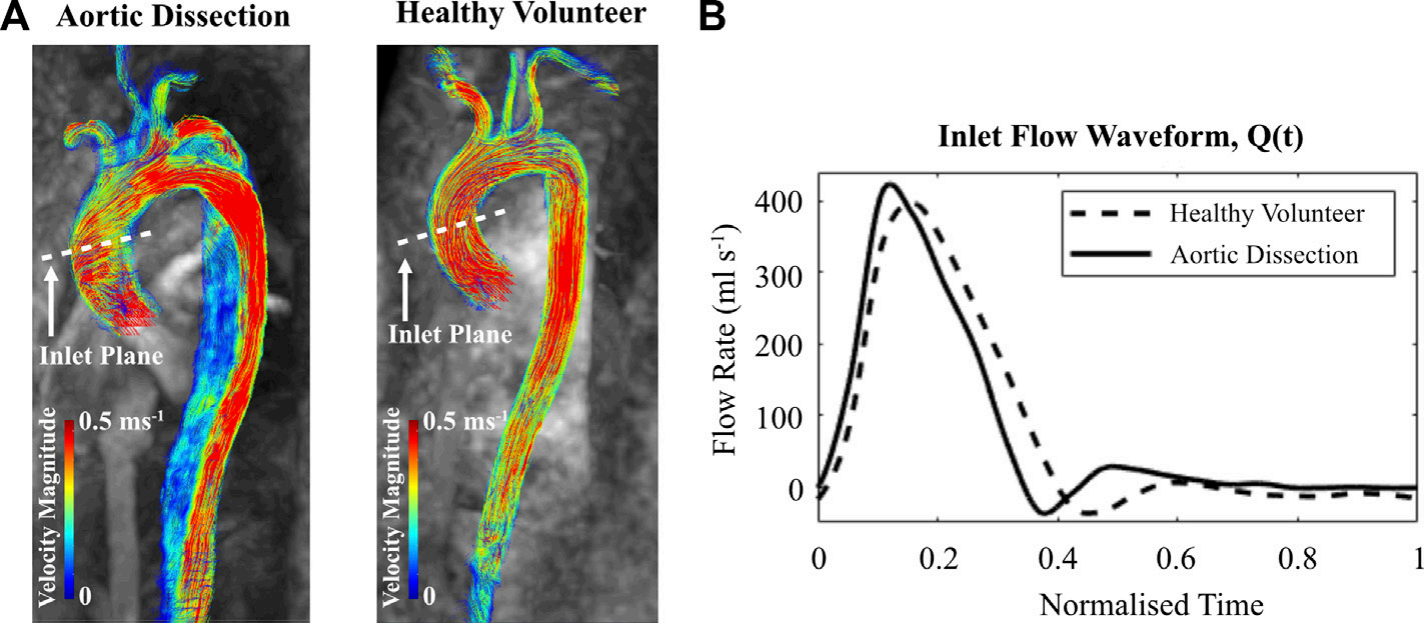
**(A)** Velocity magnitude streamlines extracted from cvi42^®^ at peak systole within the thoracic aorta of the aortic dissection patient and healthy volunteer. **(B)** 4D Flow-MRI derived flow waveform within the ascending aorta for each case.

The inlet profiles for the CFD models were extracted from the 4D Flow-MRI images. On Circle Cardiovascular Imaging software (cvi42^®^), analysis planes (n = 5) were placed at the ascending aorta of the healthy volunteer and dissected patient. In both cases, these planes were equally spaced 0.25D apart proximally and distally, with the initial plane corresponding to a location parallel to the apex of the pulmonary arch (Van-Doormaal et al., 2012). These MRI-derived flow waveforms (Figure 3) were converted to a velocity profile, interpolated to generate a constant time step size (dt = 0.001 s), and repeated for five cardiac cycles. Spatially, a uniform profile was assigned.

#### 2.4.2 Outlet branch flow waveforms

Average branch flow waveforms (Figure 1D) at the outlets were extracted from the 4D Flow-MRI data using cvi42^®^. For the right subclavian artery (RSA), right common carotid artery (RCCA), left common carotid artery (LCCA), left subclavian artery (LSA), descending aorta true lumen (DAoTL), and the descending aorta false lumen (DAoFL) five planes of analysis were placed perpendicular to the longitudinal axis of the vessel, equally spaced 0.5D apart.

#### 2.4.3 Pulse wave velocity

Arterial PWV (Figure 1D) is defined as the propagation speed of the systolic flow velocity wave front, or propagation speed of the pressure wave as it traverses the vasculature (Jarvis et al., 2022). It was calculated on cvi42^®^ for the healthy and dissected aortae. 12 planes of analysis, equally spaced throughout the aorta from the proximal ascending region to distal descending region, were retrospectively placed to calculate PWV, following the previously described methodologies in literature (Markl et al., 2010; Markl et al., 2012; Ohyama et al., 2017). The PWV of the healthy case was calculated as 7.85 ms^−1^, while the dissected case was equal to 4.38 ms^−1^. PWV is later utilized for boundary condition estimation.

#### 2.4.4 Windkessel model

The 3EWM (Eq. 5) is a hydraulic electric analogue, which models the total resistance and compliance of the peripheral vasculature to provide a dynamic description of the downstream physics (Westerhof et al., 2009; Shi and Lawford, 2011; Gharahi et al., 2016). The characteristic impedance (Z) is equal to the oscillatory pressure (P) divided by the oscillatory flow (Q), while capacitance (C) represents distal vessel wall compliance, and resistance (R) denotes the total peripheral vascular resistance (Gharahi et al., 2016).

$$\left(1+\frac{Z}{R}\right)Q\left(t\right)+CR\left(\frac{dQ\left(t\right)}{dt}\right)=\frac{P\left(t\right)}{R}+C\left(\frac{dP\left(t\right)}{dt}\right)$$ (5)

To facilitate numerical analysis, the 3EWM was discretized (Eq. 6) via the Backwards Euler finite difference method. At each terminal branch, the discretized 3EWM was coupled implicitly to the 3D numerical domain via a user defined function (UDF) in Ansys Fluent^®^. Consequently, the entirety of the vasculature distal to the 3D domain was described by a single ZRC combination for each branch. With these assigned parameter values, it was possible to calculate pressure and flow as part of the numerical solution (Spilker and Taylor, 2010).

$$P^{n+1}=\frac{\beta {P^{n}+Q}^{n+1}\left(R+Z+Z\beta \right)-{Z\beta Q}^{n}}{\left(1+\beta \right)},where\;\beta =\frac{RC}{∆t}$$ (6)

where *n* is the discrete timestep. To reduce the number of cycles required to achieve a time-periodic solution, pressure was initialized to 101 mmHg (diastolic clinical pressure) for the dissection case, and 80 mmHg (healthy diastolic reference pressure) for the healthy case (Nardi and Avrahami, 2017).

#### 2.4.5 3EWM parameter estimation

This study utilizes the arterial geometry, pulse wave velocity, and geometric scaling factors which describe the successive branching of peripheral vasculature to generate initial estimates for the 3EWM parameters (Alastruey, 2006; Xiao, 2014).

$$Z=\frac{{\rho c}_{pwv}}{A_{0}}$$ (7)

$$R=Z\left(\frac{\lambda }{{2\varphi }^{4}-\lambda }\right)$$ (8)

$$C=C_{o}\left(\frac{2\lambda \varphi^{3}}{1-2\lambda \varphi^{3}}\right),C_{o}=\frac{A_{0}l}{\rho {\left(c_{pwv}\right)}^{2}},$$ (9)

where λ = 0.68 and $\varphi =\sqrt{0.6}$ are the chosen geometric scaling factors, $c_{pwv}$ is the 4D Flow-MRI derived arterial PWV in the thoracic aorta (healthy = 7.85 ms^−1^, dissected = 4.38 ms^−1^), ρ is the blood density (1,060 kgm^-3^), $A_{0}$ is the average branch vessel cross sectional area, and l is the branch vessel length (Alastruey, 2006). For each vessel segment, the 3D model was converted to a one-dimensional (1D) geometry in the Vascular Modelling Toolkit (VMTK^©^) and the computed centerlines were utilized to obtain $A_{0}$ and l (Table 1).

**TABLE 1:** Branch vessel length and cross sectional area for the dissected and healthy aortae when converted to a one-dimensional geometry.

Branch	Branch length (m ×10^−1^)		Mean cross sectional area (m^2^ ×10^-5^)	
	Dissection	Healthy	Dissection	Healthy
RSA	1.10	0.25	4.04	7.03
RCCA	0.46	0.34	1.34	3.91
LCCA	0.80	0.66	3.35	3.46
LSA	1.48	1.35	3.05	6.75
DAoTL	1.87	2.61	12.50	43.50
DAoFL	1.85	—	25.70	—

To determine the net peripheral resistance (R_T_) required to generate a clinically accurate mean blood pressure, Eq. 10 was employed (Xiao, 2014; Xiao et al., 2014).

$$R_{T}=\frac{P_{Mean}}{{\bar{Q}}_{in}},P_{Mean}=P_{Dia}+\frac{1}{3}\left(P_{Sys}-P_{Dia}\right),$$ (10)

where ${\bar{Q}}_{in}$ is the mean inlet flow rate, P_Sys_ is the target systolic pressure, and P_Dia_ is the target diastolic pressure. For the dissected case, P_Sys_ and P_Dia_ were taken to equal 189 mmHg and 101 mmHg, respectively, which was obtained via a brachial pressure cuff measurement to complement the 4D Flow-MRI data. For the healthy case, P_Sys_ and P_Dia_ were assumed to be 120 and 80 mmHg, respectively, as pressure data was not available for the healthy volunteer (Nardi and Avrahami, 2017).

For the estimated parameters, it was then checked to ensure that:

$$\frac{1}{R_{T}}=\sum_{j=2}^{M}\frac{1}{Z^{j}+R^{j}}$$ (11)

where *M* is the number of terminal branches (excluding *j* = 1 as that is the aortic root inlet). Table 2 outlines the estimated 3EWM parameter values for each terminal branch.

**TABLE 2:** Initial estimates for the parameters of the 3EWM at each branch of the healthy and dissected models.

Branch	Windkessel parameters (estimated)					
	Z (×10^7^) [Pa s m^−3^]		R (×10^9^) [Pa s m^−3^]		C (×10^−10^) [m^3^ Pa^−1^]	
	Dissection	Healthy	Dissection	Healthy	Dissection	Healthy
RSA	11.5	6.53	2.65	1.50	3.91	0.477
RCCA	34.6	11.7	7.97	2.70	0.546	0.364
LCCA	13.9	13.3	3.19	3.05	2.36	0.621
LSA	15.2	6.80	3.5	1.57	3.98	2.5
DAoTL	3.71	1.05	0.854	0.243	20.6	31.1
DAoFL	1.81	—	0.415	—	41.9	—

### 2.5 0D-1D modelling

1D modelling was required to generate an initial estimate of pressure and flow waveforms at each branch of the thoracic aorta. For each geometry, the 1D domain was constructed from vessel centerlines of the reconstructed aortic geometries. These centerlines were partitioned into a finite number of discrete segments (N_Dissection_ = 22, N_Healthy_ = 9). For each arterial segment, the cross-sectional area and axial length were prescribed, based on the average values as computed from the centerline of that segment. The elastic wall properties were modelled via the Nektar1D empirical law, where the stiffness parameter for each vessel segment was calculated as a function of arterial PWV, blood density, and the average cross-sectional area of that segment. At each terminal branch, the estimated 3EWM BCs were coupled, thereby creating a 0D–1D model. The 4D Flow-MRI derived, patient-specific velocity waveform was applied at the inlet of this model and a fully elastic simulation was performed using the Nektar1D solver over 20 cardiac cycles (Alastruey et al., 2021). This 0D-1D simulation required ∼1 s per cardiac cycle on 2 cores (Intel^®^ Core™ i9-10900X CPU). A detailed description of the equations and numerical scheme used to solve them has been described previously in literature (Alastruey et al., 2012). The pressure and flow waveforms were extracted when the solution became time-periodic.

### 2.6 Parameter calibration

To calibrate the 3EWM parameters in order to generate patient-specific BCs, Eq. 6 was first rearranged to yield Eq. 12, thereby making the Windkessel flow rate (Q_WK_) the subject of the equation. For each terminal branch of the CFD domain, Q_WK_ was calculated as per Eq. 12, using the estimated parameters (Table 2) and the 0D-1D derived pressure waveforms. When Q_WK_ reached a time-periodic solution, the waveform over a single cardiac cycle was then compared against the *in vivo*, 4D Flow-MRI derived flow waveforms ( $Q_{inVivo}$ ) for each branch. All flow rates are presented in the units of m^3^s^−1^.

$${Q_{WK}}^{n+1}=\frac{\left(1+\beta \right)P^{n+1}+{Z\beta Q_{WK}}^{n}-\beta P^{n}}{R+Z\left(1+\beta \right)}$$ (12)

For each terminal branch, the errors ( $\varepsilon_{j}$ ) present between the clinical ( $Q_{inVivo}$ ) and simulated (Q_WK_) data points was calculated as per Eq. 13:

$$\varepsilon_{j}=\sum_{i=1}^{T}{\left(Q_{WK}\left(t_{i}\right)-Q_{inVivo}\left(t_{i}\right)\right)}^{2}$$ (13)

where *T* is the duration of a single cardiac cycle, j=1,2,…,M where *j* is the related terminal branch and *M* is the total number of branches, and $t_{i},i=1,2,$ …, T are the measurement time points, where dt = 0.001 s.

**FIGURE 4:**
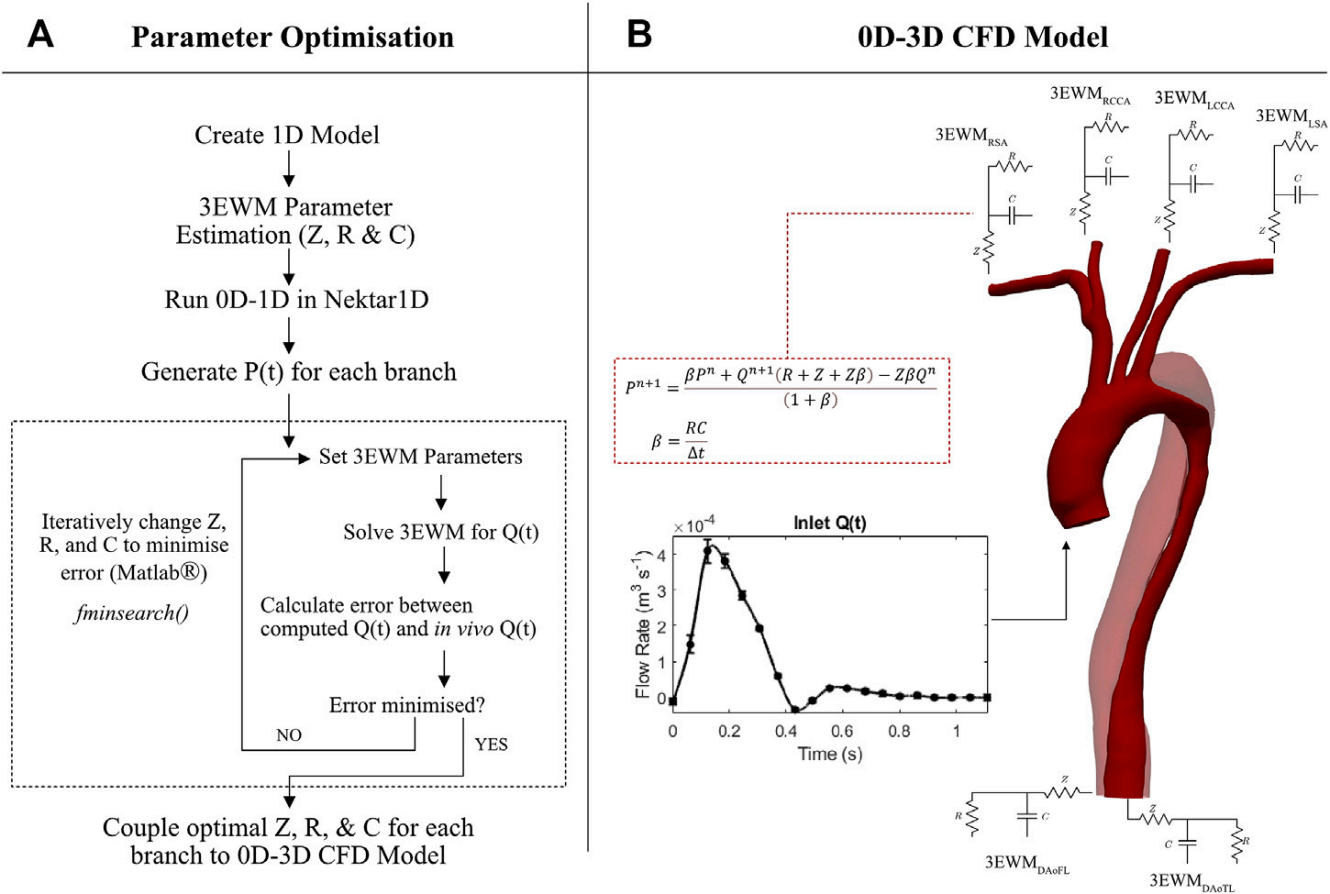
**(A)** Detailed flow diagram of the methodology used to calibrate impedance (Z), resistance (R), and compliance (C) of the 3EWMS BCs. **(B)** 0D–3D CFD model set-up, where each branch was coupled with a 3EWM. At the inlet, a 4D Flow-MRI derived flow waveform was converted to a parabolic velocity profile. The discretised 3EWM equation describes the pressure (P) and flow (Q) relationship at each branch, where n denotes the current iteration.

The Windkessel parameters were then iteratively changed to minimise ε by employing the *fminsearch* routine via an in-house Matlab^®^ script at each branch of the thoracic aorta (Figure 4). Therefore, the aim of the calibration process was to find a parameter combination at each branch which resulted in a flow waveform which was most representative of the clinical 4D Flow-MRI data. This utilises a direct search method (Nelder-Mead simplex algorithm) such that the simplex hosts n + 1 points, where n = 3 is the dimension of the problem (Lagarias et al., 1998). The initial Windkessel parameter estimates formed the initial simplex, whereafter the routine implemented a series of transformations (e.g., reflection, expansion, etc.) such that the final simplex hosts the best-fitting parameter values that correspond to the smallest error ( $\varepsilon_{j}$ ), within a user-defined tolerance limit of 10^−6^ for the 3EWM parameters and 10^−8^ for $\varepsilon_{j}$ . Finally, the calibrated, patient specific 3EWM BCs were coupled to the 3D model (Figure 4).

## 3 Results

Results are presented below in a series of tables and figures, describing the calibrated 3EWM parameters and the CFD-derived perfusion distribution, pressure, TAWSS, and OSI. These results demonstrate the application of these calibrated BCs on a healthy volunteer and clinical patient with aortic dissection as proof-of-concept examples. We show that our methodology yields a perfusion distribution which very closely matches *in-vivo* 4D Flow-MRI-derived data, and physiologically accurate near-wall hemodynamics.

### 3.1 3EWM BC calibration (0D—Matlab^®^)

To create patient-specific BCs, a total of 18 parameters were calibrated for the dissected case, and 15 for the healthy case. This was an iterative process, requiring 20 cardiac cycles per iteration, and 100–120 iterations per parameter combination. To complete this process with the combination of reduced order, computationally efficient 0D and 1D models described in this study, it required only 3.5 min per branch, on average. The final 3EWM parameters which were calculated after completion of the simplex-based calibration are presented in Table 3.

**TABLE 3:** Final 3EWM parameter combination for each branch of the dissected and healthy models upon completion of the calibration process.

Branch	Windkessel parameters (calibrated)					
	Z (×10^7^) [Pa s m^−3^]		R (×10^9^) [Pa s m^−3^]		C (×10^−10^) [m^3^ Pa^−1^]	
	Dissection	Healthy	Dissection	Healthy	Dissection	Healthy
RSA	4.27	0.137	3.70	1.82	1.07	3.65
RCCA	3.85	0.296	3.53	3.22	1.08	1.38
LCCA	3.98	0.303	3.44	3.82	0.632	1.50
LSA	3.71	0.842	3.94	1.94	1.22	4.20
DAoTL	0.204	0.391	0.290	0.177	11.2	46.6
DAoFL	1.64	—	7.92	—	5.80	—

**FIGURE 5:**
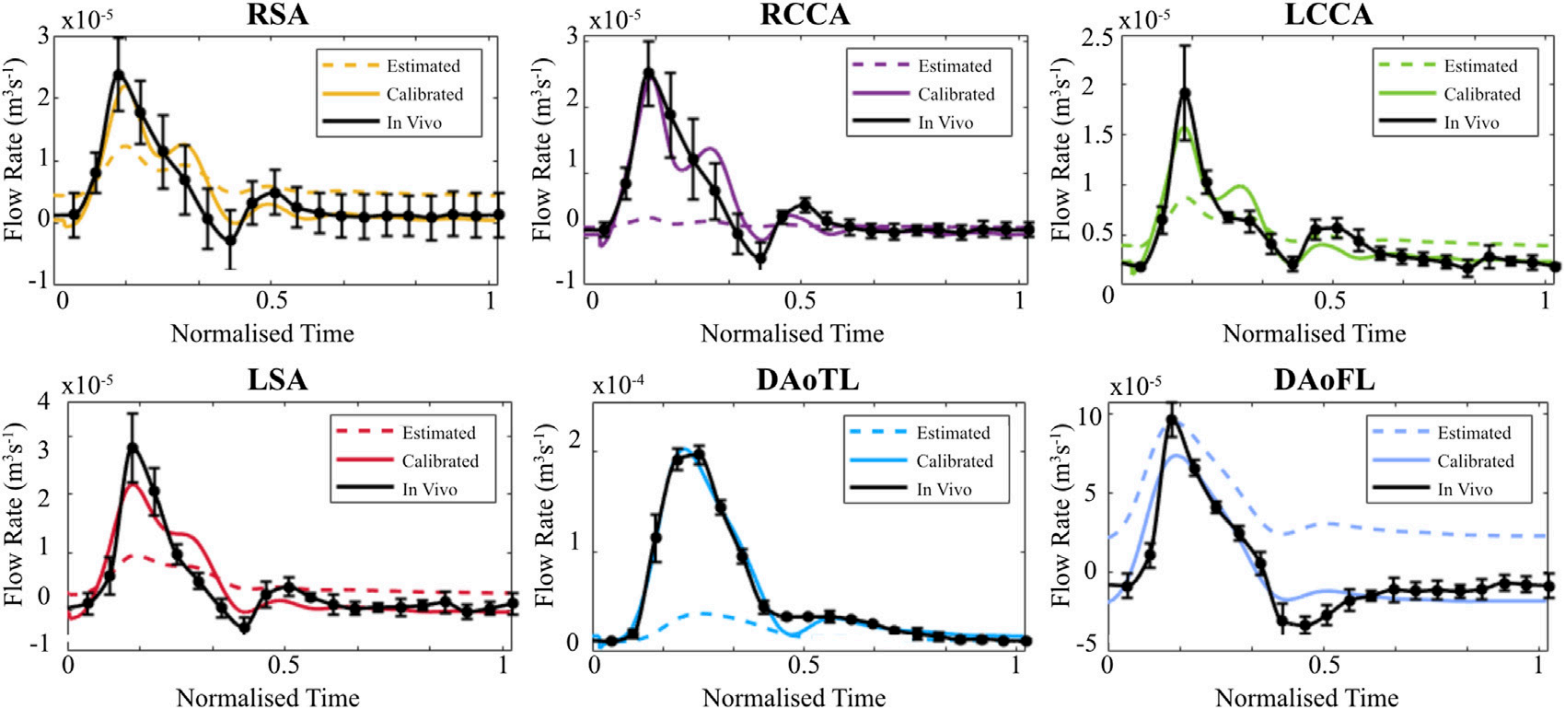
Flow waveforms for each branch of the thoracic aorta for the patient with an AD as calculated via the 0D 3EWM before (dashed colored lines) and after (solid colored lines) calibration of Z, R, and C. The *in vivo* 4D Flow-MRI derived waveforms are shown in black (mean ± standard deviation).

**FIGURE 6:**
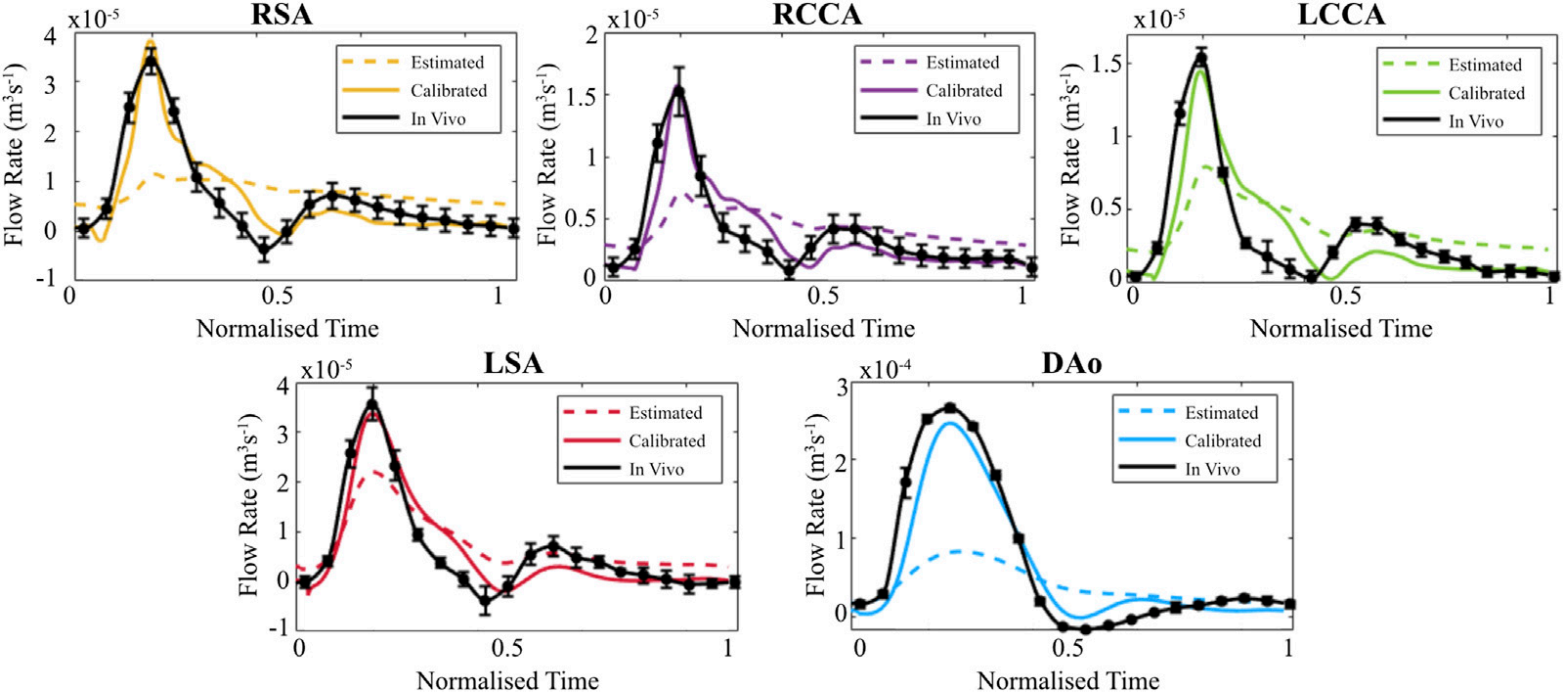
Flow waveforms for each branch of the thoracic aorta for the healthy volunteer as calculated via the 0D 3EWM before (dashed colored lines) and after (solid colored lines) calibration of Z, R, and C. The *in vivo* 4D Flow-MRI derived waveforms are shown in black (mean ± standard deviation).

When evaluated within the 0D Matlab^®^ framework, the calibrated parameters yield a more accurate and physiologically relevant flow waveform for each branch of the thoracic aorta for the AD patient (Figure 5) and healthy volunteer (Figure 6). In both cases, the calibrated parameters dramatically reduced the cumulative least squares difference (LSD) error between the computed and *in vivo* data. Regarding the dissected case, the error for the RSA, RCCA, LCCA, LSA, DAoTL, and DAoFL, was reduced with respect to the estimated parameters by 75.1%, 88.6%, 74.4%, 74.5%, 98.9%, and 92.2%, respectively. In the healthy case, these cumulative LSD errors were reduced by 81.8%, 75.3%, 56.2%, 58.4% and 88.8%, respectively.

### 3.2 0D–3D CFD model

#### 3.2.1 Perfusion distribution

**FIGURE 7:**
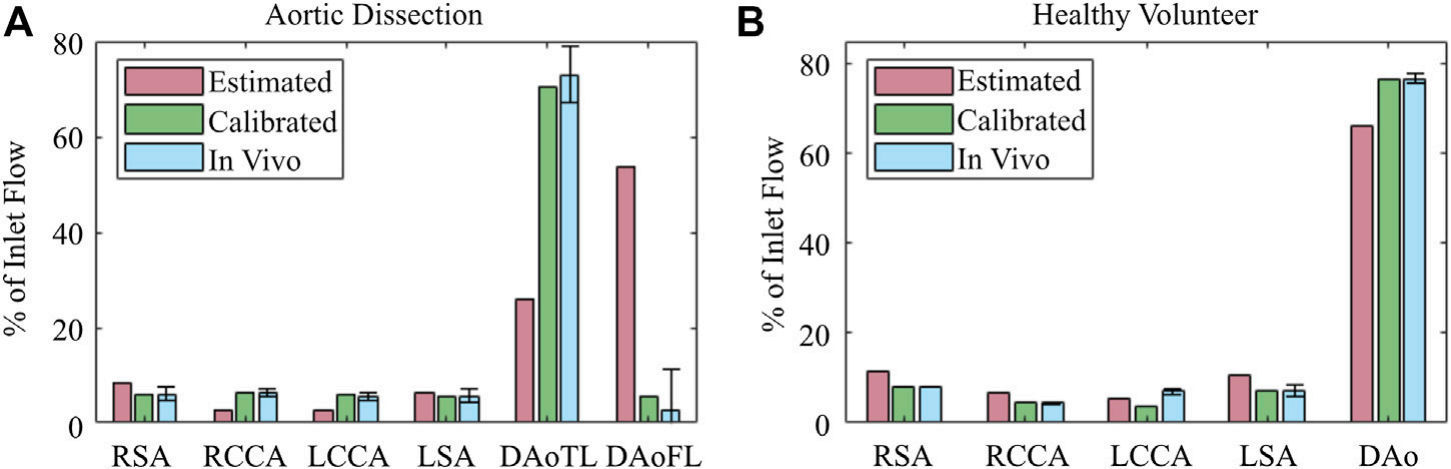
Blood flow perfusion distribution throughout the 0D–3D CFD model of the **(A)** aortic dissection and **(B)** healthy volunteer, before (red) and after (green) BC calibration, in comparison with (blue) *in vivo* 4D Flow-MRI obtained data. Error bars represent mean ± standard deviation.

BC calibration substantially improved the net perfusion distribution (Figure 7) throughout the aorta in both the healthy and dissected cases. This was particularly evident in the dissection model, where calibration of the 3EWM parameters reduced the error in the TL from 47.0% to 2.5%, and in the FL from 50.8% to 2.87% with respect to the *in vivo* data. At the other branches, and in the healthy model, the error reductions were less dramatic, though still presented an improvement. The exception to this was the LCCA branch of the healthy model, where calibration increased the error with respect to *in vivo* data.

#### 3.2.2 Arterial pressure

The choice of BCs impacted the pressure at each branch of the 0D-3D CFD models (Table 4). Generally, calibration of the 3EWM parameters tended to dampen the pulse pressure as a result of an increased diastolic pressure and decreased systolic pressure when compared to the estimated parameters.

**TABLE 4:** Systolic pressure, diastolic pressure, pulse pressure, and mean arterial pressure obtained from 0D–3D CFD models of the aortic dissection and healthy volunteer. For each variable, mean ± standard deviation was calculated by averaging across the supra-aortic branches and descending aorta. Reference values for the healthy volunteer were obtained from literature (Nardi and Avrahami, 2017).

	Estimated BCs	Calibrated BCs	*In Vivo* clinical data
Dissection			
Systolic pressure (mmHg)	189 ± 2.66	167 ± 10.5	189
Diastolic pressure (mmHg)	103 ± 0.05	117 ± 0.59	101
Pulse pressure (mmHg)	86.0 ± 2.66	50.0 ± 10.5	88
Mean arterial pressure (mmHg)	130 ± 0.93	130 ± 4.26	130
Healthy volunteer	**Estimated BCs**	**Calibrated BCs**	**Literature**
Systolic pressure (mmHg)	151 ± 4.85	125 ± 5.30	120
Diastolic pressure (mmHg)	47.6 ± 0.81	51.5 ± 0.78	80
Pulse pressure (mmHg)	103 ± 4.91	80 ± 5.36	40
Mean arterial pressure (mmHg)	82.1 ± 1.88	76.0 ± 1.75	93.3

#### 3.2.3 TAWSS, OSI, and pressure distribution

**FIGURE 8:**
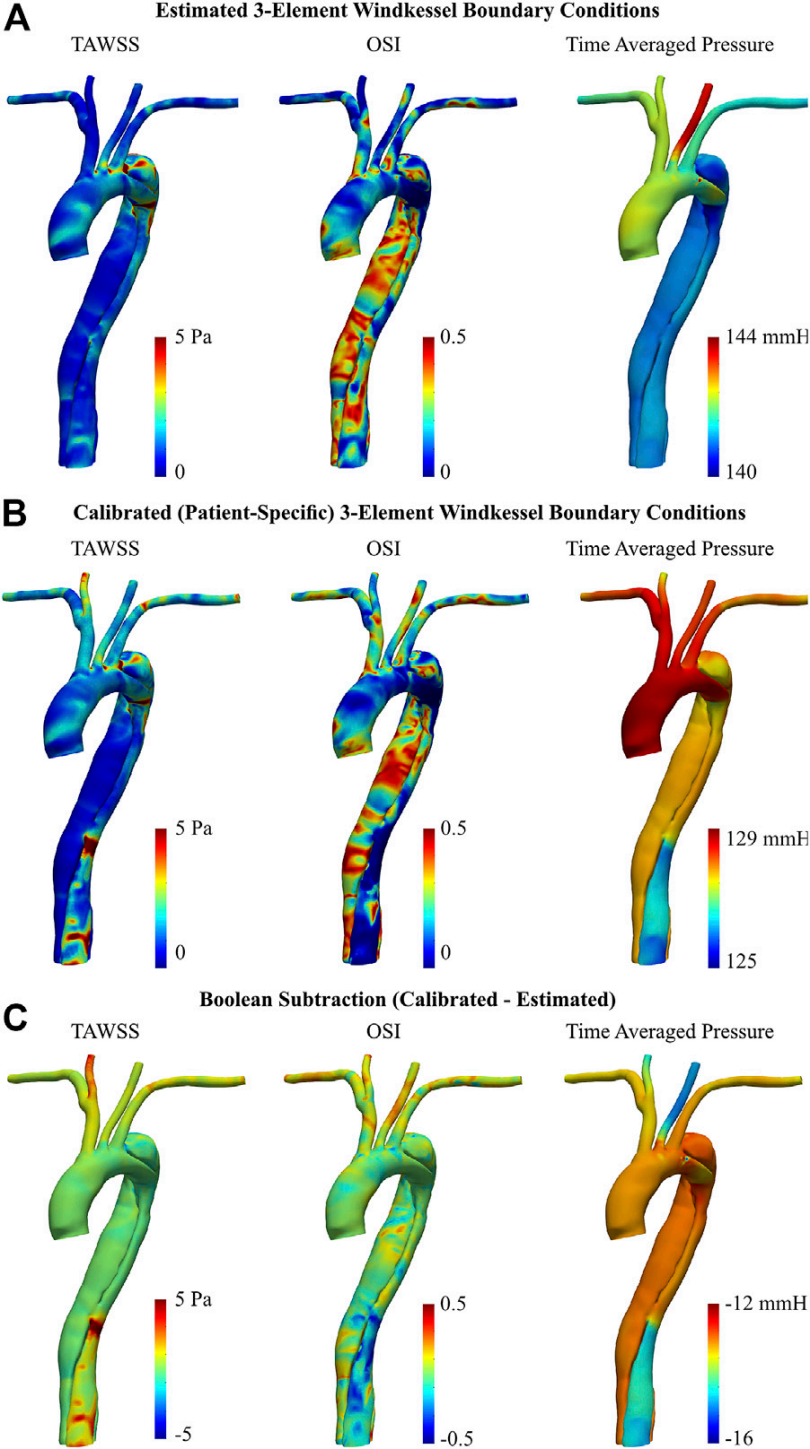
TAWSS, OSI, and time averaged pressure of dissected aorta, obtained via 0D–3D CFD simulation using **(A)** estimated, and **(B)** calibrated 3EWM BCs. **(C)** Boolean difference (Distribution_Calibrated_−Distribution_Estimated_) throughout the models.

Near-wall hemodynamics are affected both by the arterial geometry and applied boundary conditions (Alastruey et al., 2012; Gijsen et al., 2019). Notably, the effect of smoothing the reconstructed geometries as outlined in the methodology will not significantly influence these results (Celi et al., 2021). Figure 8 illustrates the distribution of TAWSS, OSI, and pressure distribution in the AD case before and after BC calibration. In both the estimated and calibrated models, regions of elevated TAWSS were identified immediately distal to each supra-aortic branch ostia. With the estimated 3EWM BCs, other elevated regions of TAWSS were localized to the primary tear and the distal region of the aortic arch within the proximity of the secondary tear. Further, TAWSS was minimal in both the TL and FL with the estimated BCs (Figures 8A, B), and there was negligible difference in the magnitude of TAWSS between these lumens. After BC calibration, TAWSS was reduced in the bulbous FL of the aortic arch by up to 2.04 Pa and was increased at the TL region immediately distal to the descending secondary tear by up to 14.4 Pa (Figure 8C). Additionally, calibration increased the TAWSS throughout the supra-aortic branch vessels. Finally, calculation of a surface integral at each tear region revealed that BC calibration can alter TAWSS by 14.5% at the primary tear to 46.6% at the distal secondary descending tear.

Regarding OSI, the spatial distribution throughout the ascending aorta and supra-aortic branches remained generally unchanged after BC calibration, though there was a general increase in magnitude. There was, however, a marked qualitative and quantitative difference in the OSI in the descending TL and FL between the estimated and calibrated models, most notably in the distal region. After calibration, OSI within the TL was reduced by up to 0.49, and OSI within the FL was increased by up to 0.43 (Figure 8C). Considering OSI is a non-dimensional quantity bounded between 0 and 0.5, these are substantial changes. Finally, BC calibration had a marked effect on the average pressure distribution, reducing pressure by as much as 16 mmHg. This BC calibration also resulted in a more uniform distribution of pressure throughout the supra-aortic branch vessels.

**FIGURE 9:**
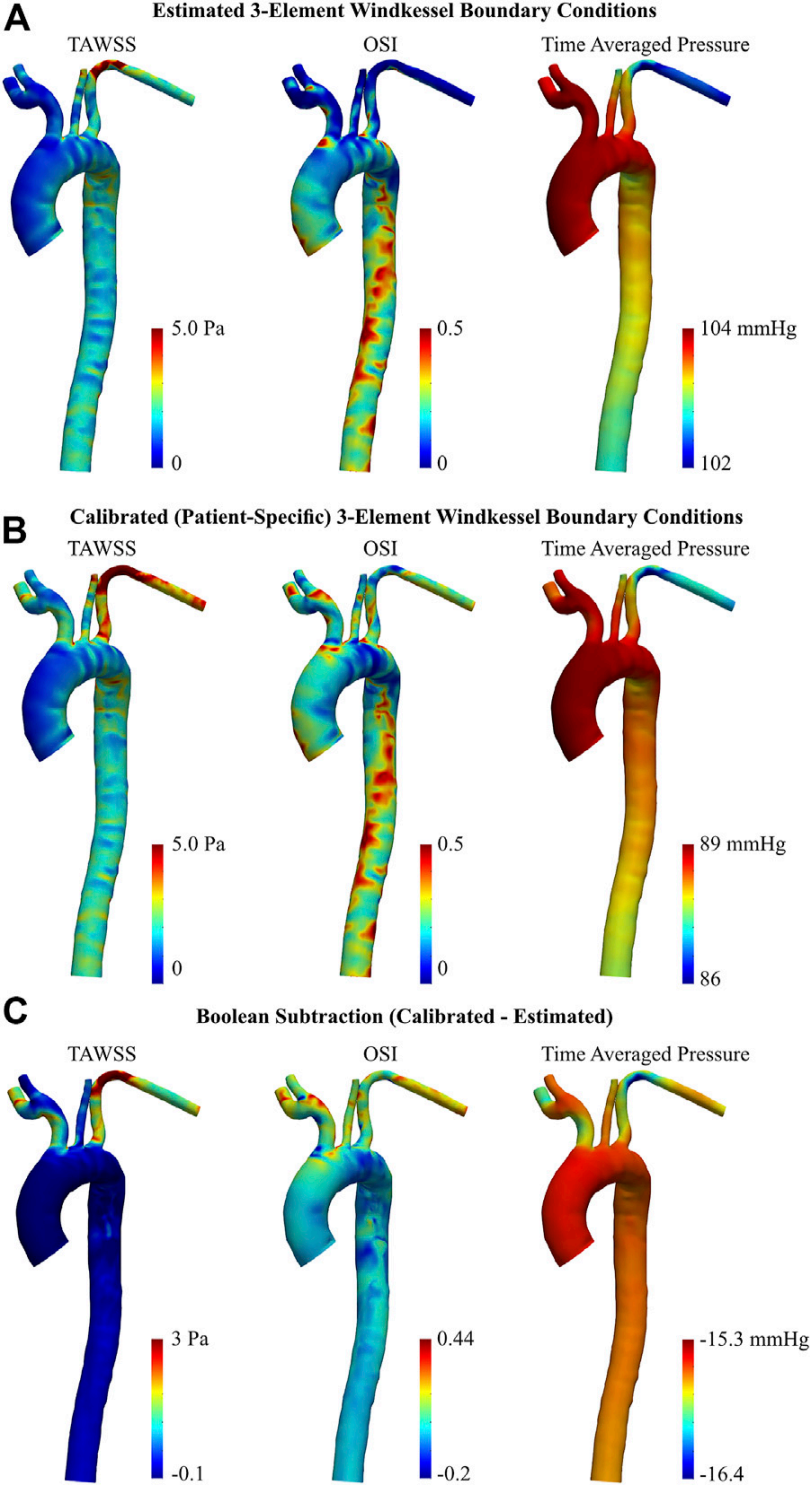
TAWSS, OSI, and time averaged pressure of healthy aorta, obtained via 0D-3D CFD simulation using **(A)** estimated, and **(B)** calibrated 3EWM BCs. **(C)** Boolean difference (Distribution_Calibrated_−Distribution_Estimated_) throughout the models.

Regarding the healthy volunteer, TAWSS was substantially reduced in the LSA following BC calibration, by up to 5.05 Pa (Figure 9C). To a lesser degree, a subsequent increase was apparent around the base of the brachiocephalic and RSA arteries. Throughout the main body of the aorta however, there were minimal differences in TAWSS between the estimated and calibrated models.

BC calibration maintained the spatial pattern of OSI throughout the entire aorta, primarily altering the magnitude (Figures 9A, B). Notably, the calibrated model showed regions of elevated OSI throughout the supra-aortic branches and in the region immediately proximal to the brachiocephalic artery when compared to the estimated case (Figure 9C). Conversely, throughout the descending aorta, BC calibration had a reduced effect, altering OSI by ∼0.2 (Figure 9C). Time-averaged pressure was reduced by 15.3–16.4 mmHg following BC calibration.

#### 3.2.4 CFD vs. 4D Flow-MRI blood velocity

**FIGURE 10:**
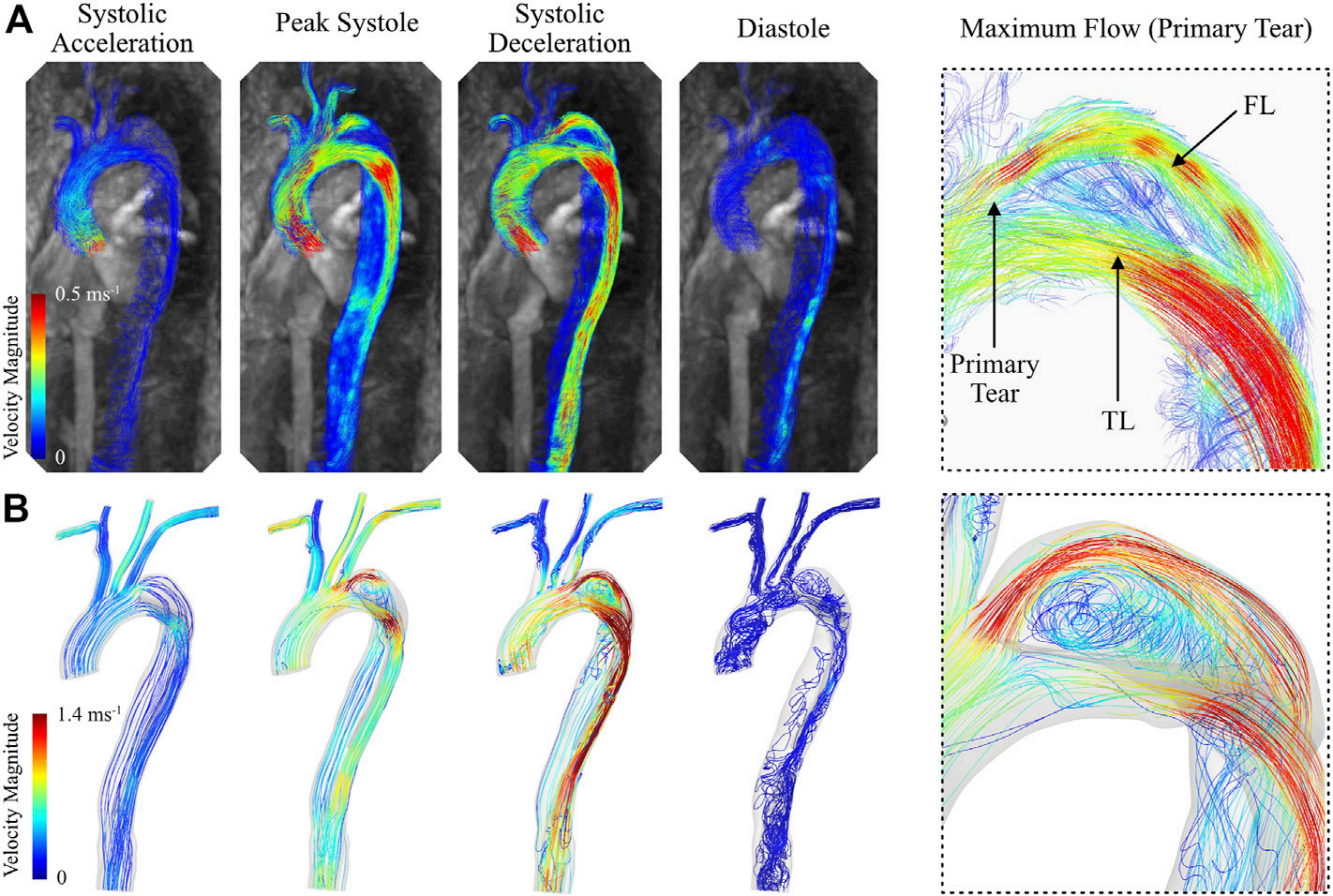
Instantaneous blood velocity magnitude streamlines of the type B thoracic aortic dissection obtained from **(A)** 4D Flow-MRI and **(B)** CFD modelling at systolic acceleration, peak systole, systolic deceleration, and diastole. Also visible is the maximum velocity within the FL immediately distal to the primary tear, as extracted from both CFD and 4D Flow-MRI.

The calibrated 0D–3D CFD model of the type B aortic dissection qualitatively captured the complex flow regime within the TL and FL of the thoracic aorta when compared against *in vivo* data (Figure 10). For example, the CFD model successfully depicted the region of flow recirculation within the false lumen of the aortic arch between the primary and secondary tear. Additionally, the model captured regions of high flow at the primary tear, the superior boundary of the FL, and through the TL at the distal section of the aortic arch. Quantitatively, CFD analysis resulted in overestimation of blood velocity during the systolic cardiac phases, and a subsequent underestimation (increased flow reversal) during the diastolic phases. Therefore, while the net flow through each branch was successfully calculated, there are discrepancies in the instantaneous velocity magnitude between the CFD models and *in vivo* data. This was most apparent during peak systole and systolic deceleration.

## 4 Discussion

Patient-specific branch flow rates are not often prescribed as BCs in CFD models. This is because the prescription of such profiles can lead to inaccurate pressure calculations and tend to overprescribe the model for any future parametric analysis (Vignon-Clementel et al., 2006). Thus, the 3EWM model is ubiquitously used instead for the prescription of physiologically relevant BCs (Spilker and Taylor, 2010; Zhou et al., 2019). This study outlines a methodology to calibrate these 3EWM models to generate patient-specific BCs and proof-of-concept examples illustrate that the resultant CFD models have the capability to elucidate patient-specific hemodynamics which are consistent with previous literature and clinical measurements. It is possible that, in the future, CFD models calibrated from 4D Flow-MRI blood flow data may be utilized to derive hemodynamic parameters which cannot otherwise be extracted from *in vivo* data and hence may contribute to clinical decision making. For example, such models may highlight a potentially high-risk rupture site of the false lumen.

### 4.1 4D Flow-MRI processing

Literature shows that 4D Flow-MRI quantification of non-laminar blood flow shows good correlation against the reference gold standard (2D phase contrast MRI) (Gabbour et al., 2013; Bollache et al., 2016). Thus, calibration of the 3EWM BCs against the 4D Flow-MRI derived flow rates was deemed appropriate. PWV is a well-established measurement which is positively associated with aortic stiffness and is readily obtained from 4D Flow-MRI (Kröner et al., 2011; Ghosh et al., 2018). Conventionally, MRI-derived PWV is limited to 2–4 pre-defined planes of analysis (Shahzad et al., 2019; Hout et al., 2022). Since 4D Flow-MRI was utilized in this study, it was simple to retrospectively place 12 analysis planes throughout the entire length of the thoracic aorta, thereby improving the reliability of the measurement (Markl et al., 2010; Markl et al., 2012). The healthy volunteer exhibited a PWV of 7.85 ms^−1^, further demonstrating the validity of this approach as literature suggests the median PWV of a large cohort of healthy individuals (n = 3071) is 7.2 ms^−1^ (Ohyama et al., 2017).

### 4.2 BC calibration

The combination of 0D and 1D models utilized in this study facilitated rapid, computationally efficient parameter calibration. To determine the most effective calibration algorithm on Matlab^®^, multiple different multivariate solvers (*fminsearch*, *fmincon*, *fminunc*) were tested. It was confirmed that *fminsearch* consistently minimised the error between the computed and *in vivo* flow rate to the greatest degree. To further demonstrate the reliability of this method, the initial parameter input estimates were manually and individually perturbed by a factor of 0.125, 0.25, 0.5, 2, 4, and 8 to determine the sensitivity of the model to local variations. Even with these perturbations, the solution converged on the same parameter combination each time. The effectiveness of this approach is evident from the reduction in cumulative LSD error between the computed and *in vivo* flow rates by 74.4%–98.2% and 56.2%–88.8% in the dissected and healthy case, respectively. Notably, the calibration process also satisfied the requirement of R>>Z in all branches (Grinberg and Karniadakis, 2008).

Small discrepancies remained between the computed and *in vivo* waveforms after calibration. It is possible that this was partly a result of a non-patient-specific pressure waveform generated by the 0D–1D models since the calibration process was sensitive to changes in the initial pressure waveform. It was not possible to validate these waveforms in the absence of time-resolved clinical pressure data. Discrepancies between the *in vivo* and calibrated computed flow waveforms may also be explained as follows. Firstly, it may not be possible to exactly fit a relatively simple function like Eq. 12 to the complex *in vivo* flow waveforms. Secondly, it is possible that the algorithm to minimize the error function during calibration may fall within a local minimum as opposed to the global minimum, meaning the solution may not be unique. However, the 3EWM parameter combination which produces a global minimum may include values so different from the initial estimates that they are not physiologically relevant (i.e., negative values, impedance greater than resistance, extremely high or low value for one or more parameter). The parameter combinations presented within this study are, however, known to be within the physiological range.

### 4.3 0D–3D CFD model

The combination of 4D Flow-MRI and coupled 0D–3D CFD models produced a comprehensive picture of the complex flow regime and near-wall hemodynamics in both the healthy and dissected aortae.

#### 4.3.1 Perfusion distribution

The impact of BC calibration was more significant in the dissected model when compared to the healthy case, indicating that the initial geometry-based estimates failed to predict the complex and highly individualized flow regime as a result of aortic pathology such as AD (Alastruey, 2006; Xiao, 2014). This is because the severity, location, number of intraluminal tears, and overall geometry of the pathology varies significantly on a case-by-case basis. For example, the estimated BCs demonstrated a tendency to overestimate flow at the outlet of the FL due to the increased vessel diameter and the expectation of decreased hydraulic resistance. As a result of continuity, this caused a subsequent underestimation of flow within the TL. The BC calibration process, however, rectified this issue to generate a more physiologically accurate perfusion distribution which is comparable to the *in vivo* data and previous studies (Liu et al., 2018). This improvement is important since blood flow rate and flow regime are key factors which influence the expansion of the FL, the successive collapse of the TL, and degree of peripheral organ malperfusion in AD (Alimohammadi et al., 2014; Crawford et al., 2016; Liu et al., 2018). Further, the calibrated models yield increased flow through the supra-aortic branch vessels, which was also observed in the 4D Flow-MRI data. This observation is both clinically relevant and expected, as patients with AD often experience this increase in flow due to an elevated hydraulic resistance in the descending aorta (Alimohammadi et al., 2015). The effect of BC optimization was less pronounced in the healthy case, though an improvement in the perfusion distribution was still notable, especially through the descending aorta.

#### 4.3.2 TAWSS and OSI

Accurate portrayal of near-wall hemodynamics is fundamental as the 3EWM parameters play a crucial role in the regulation of the arterial structure, and the initiation and progression of disease (Ku, 1997; Zaman et al., 2009). For example, initiation of the primary tear of an AD often occurs immediately distal to the LSA due to flow disturbance within this region (Callaghan and Grieve, 2018). Notably, this is where the primary tear is located for the AD patient in this study.

In both aortae, the TAWSS exhibited a heterogeneous spatial distribution, as expected (Callaghan and Grieve, 2018). In the healthy case, there were localized, elevated regions of TAWSS (∼5 Pa) immediately distal to the supra-aortic branch ostia, which is spatially consistent with previous studies (Shahcheraghi et al., 2002; Callaghan and Grieve, 2018). As there was no pathology in the geometry of the healthy individual, the estimated BC models were well equipped to predict the spatial distribution of TAWSS and OSI. Thus, calibration in the healthy case served mainly to alter the magnitude of near-wall hemodynamics.

In the case of the AD, calibration exhibited a more marked effect on both the magnitude and spatial pattern of these parameters. In the calibrated model, TAWSS in the TL was increased due to increased flow in the relatively narrow lumen, which is consistent with literature (Tse et al., 2011; Liu et al., 2018). Further, the TL also experienced a reduction in OSI which is indicative of an increased degree of unidirectional flow, as expected (Kazakidi et al., 2011). Conversely, OSI was increased in the FL, indicative of a more chaotic flow regime. Analysis of the 4D Flow-MRI images confirmed that both of these findings are consistent with the *in vivo* data as the regurgitation fraction was low in the TL and high in the FL. These findings are also consistent with previous studies (Alimohammadi et al., 2014; Xu et al., 2018). Further, regions of considerably elevated TAWSS following BC calibration were observed at each intraluminal tear, and were a result of jet flow through the narrow opening (Tse et al., 2011; Alimohammadi et al., 2014). Notably, the estimated model failed to capture this in the region of the secondary tear in the descending aorta but was rectified following BC calibration. These findings indicate the importance of BC calibration to generate patient-specific CFD models and clinically relevant results. This is particularly important in cases of AD, where the flow regime is highly dependent on the dissection location and severity (Chen et al., 2013; Liu et al., 2018; Bonfanti et al., 2019).

#### 4.3.3 Pressure

In the presence of an AD with small secondary tears, one would expect a large pressure difference between the TL and FL, with higher pressures in the former (Rudenick et al., 2013; Bonfanti et al., 2019). However, the AD presented in this study has two large tears, one being the primary tear at the LSA (17 mm) and the other being the secondary tear in the distal descending aorta (19 mm) (Rudenick et al., 2013; Evangelista et al., 2014). In the presence of such large tears, there is a tendency for pressure within the TL and FL to equalize, which was demonstrated throughout the proximal aorta in this study (Rudenick et al., 2013). A further reduction of velocity within the FL and increase in regurgitation then occurs distal to the descending secondary tear. This results in a higher pressure in the FL compared to the TL in the distal aorta which is consistent with previous literature (Alimohammadi et al., 2014; Liu et al., 2018; Xu et al., 2018). Capturing this pressure gradient is essential as it can influence expansion of the FL and compression of the TL, resulting in a potential hypertensive crisis or at worst, fatal rupture of the aortic wall (Chen et al., 2013; Alimohammadi et al., 2014). This was only captured after BC calibration.

In the AD case, the estimated BCs more accurately capture the magnitude of systolic and diastolic blood pressure as obtained from a brachial cuff measurement. However, they induce an almost identical systolic and diastolic pressure at all branch outlets, which contradicts previous literature (Tse et al., 2011; Chen et al., 2013; Bonfanti et al., 2019). Conversely, prescription of the calibrated BCs dampens the pulse pressure (∼41 mmHg) and reduces systolic blood pressure, thereby creating a discrepancy between the computed and clinical data. This can be explained due to the well-known phenomena of pulse pressure amplification, where arterial stiffness and therefore systolic blood pressure increases from the central aorta towards the peripheral brachial artery (Wykretowicz et al., 2012; McEniery et al., 2014). Literature suggests that in the age range of 50–60 years old, the central aortic pressure should be 9 ± 6 mmHg lower than the observed brachial pressure. In this study, the decrease in central systolic blood pressure and pulse pressure after BC calibration, when compared to the brachial cuff measurement, accounts for this phenomenon and therefore yields a more physiologically relevant magnitude. After BC calibration, diastolic pressure also increased, though literature suggests it should remain relatively constant with regards to the brachial measurement (Safar et al., 2018). Future studies will include the 1D model within the calibration process and compliant walls within the CFD model to investigate the effect of these added components in the calculation of the final diastolic pressure.

In the healthy case, prescription of these estimated BCs significantly overestimates systolic blood pressure by ∼31 mmHg, and underestimates diastolic pressure by ∼32 mmHg. After calibration, these errors are reduced to ∼5 mmHg and ∼29 mmHg, respectively. Without modelling aortic wall compliance in the 3D domain however, it is not possible to determine the exact consequence of BC calibration (Liu et al., 2018; Bonfanti et al., 2019).

#### 4.3.4 CFD vs. 4D Flow-MRI blood velocity

While the CFD models accurately captured the net flow throughout the aorta in the healthy and dissected cases, the BC calibration methodology resulted in discrepancies in instantaneous velocity magnitude. This was due in part to the calibration methodology, and in part due to the fundamental differences between the *in vivo* data and CFD models. The calibration process was performed using a combination of 1D and, primarily, 0D modelling and optimization. The reason for this was to avoid the prohibitive computational cost associated with 3D modelling, as an extensive number of iterations were required. Consequently, the 3EWM parameters which were calibrated within these lower order models produced slightly different results when coupled to the 3D CFD model. Future work will aim to improve the calibration process to capture the instantaneous velocity magnitude of blood more accurately by integrating the Nektar1D solver into the calibration process to include the effects of the 1D spatial domain, wave reflections, and vessel wall compliance.

Due to the inherent differences between CFD modelling and 4D Flow-MRI, discrepancies in blood velocity are unavoidable (Misaki et al., 2021; Cherry et al., 2022; Shahid et al., 2022). There are several reasons for this. Firstly, the rigid wall CFD models did not account for arterial compliance, meaning the cross-sectional area of the lumen could not expand to accommodate the increased flow during systole (to reduce blood velocity) or contract during diastole (to increase blood velocity). In contrast, the 4D Flow-MRI derived velocities account for this compliance. Further, the CFD spatiotemporal resolution is very high compared to the relatively coarse spatial and temporal resolution of 4D Flow-MRI, which is known to result in blood velocity differences, primarily in regions of elevated flow (Misaki et al., 2021; Shahid et al., 2022). Further, the 4D scan sequence utilized to obtain the retrospective data employed a spatial resolution of 3.6 mm × 2.4 mm × 2.6 mm which is coarser than the minimum resolution suggested in literature (1.5 mm × 1.5 mm × 1.5 mm), resulting in increased data interpolation to calculate *in vivo* blood velocity (Misaki et al., 2021). Additionally, the scan sequence employed an anisotropic spatial resolution, indicating that the final *in vivo* results are directionally dependent unlike the CFD models (Misaki et al., 2021).

## 5 Limitations and future work

It is acknowledged here that there are some limitations in this study. Only one healthy and one AD case is considered, which were intended as proof-of-concept examples and not as a clinical study. Through these cases it is demonstrated how the novel methodology contributes towards the development of patient-specific BCs for arterial CFD models. In the future, this methodology will be implemented on a larger cohort to evaluate the distribution of hemodynamics on healthy and diseased patients.

The 0D-1D model used to generate the initial pressure waveform could not capture the complex secondary flows, regurgitation, and regions of recirculation which are present around the intraluminal tears (Alastruey et al., 2012; Xiao et al., 2014). In the dissected case, these uncertainties were likely magnified around the intraluminal tears.

After the initial pressure and flow waveforms were estimated with 0D-1D modelling, the iterative BC calibration was performed using only a 0D solver. Future work will integrate the 0D-1D model with the Nelder Mead (*fminsearch*) algorithm to ensure wave reflections and spatial variation in the arterial geometry are accounted for during calibration. Additionally, viscoelastic wall properties will be utilized in place of the elastic wall assumption to generate more physiologically accurate pressure waveforms. Further, the spatial and temporal resolution of the retrospective 4D Flow-MRI data was limited, likely introducing an intrinsic error in the BC calibration process. Future work will require a prospective 4D Flow-MRI scan with improved spatiotemporal resolution and a multi-VENC sequence to capture blood flow more readily within the supra-aortic branches and FL.

To reduce computational demand, a rigid wall assumption was utilized for the 0D-3D CFD models. However, it is known the aorta distends to accommodate increases in blood volume throughout the cardiac cycle (Alastruey et al., 2012). In cases of AD, this wall motion becomes more important (Bonfanti et al., 2019). Future work will therefore include fluid structure interaction (FSI) to replicate vessel wall compliance. Additionally, the simulations performed in this study were restricted to one node on the high-performance computing cluster, resulting in relatively long simulation times. Future work will expand upon these proof-of-concept results to generate high-fidelity CFD models which shall be simulated across multiple parallel nodes.

Spatially, a uniform inlet profile was prescribed in the absence of decomposed x, y, and z velocity magnitudes from the *in vivo* data. Though literature suggests an idealized paraboloid is sufficient in the absence of such data, it still fails to capture the effect of the aortic valve on blood flow through the aortic root. Where applicable, future studies will extract the 3D spatial inlet profile from PC-MRI to overcome this issue.

## 6 Conclusions

To create high-fidelity arterial CFD models, it is essential to prescribe accurate BCs. This study outlines a novel approach for the calibration of patient-specific, physiologically relevant 3EWM BCs based on *in vivo* flow waveforms obtained from retrospective 4D Flow-MRI. Based exclusively on non-invasive measurements, the arterial impedance, resistance, and compliance parameters were rapidly calibrated in a computationally efficient, reduced order framework. This calibration was particularly important in cases of AD to elucidate the intricate crossflow between the TL and FL and capture flow phenomena in the highly individualized morphological features of the pathology. Following parameter calibration, blood flow was modelled in a coupled 0D–3D numerical framework, yielding physiologically relevant hemodynamics in proof-of-concept examples. These CFD models exhibited a perfusion distribution which closely matches the clinical data, and offer promising preliminary results regarding OSI, TAWSS, and pressure distribution. By enhancing the information obtained from 4D Flow-MRI, this combination of CFD and medical imaging yields a comprehensive understanding of patient-specific aortic hemodynamics.

## Data Availability

The original contributions presented in the study are included in the article/Supplementary Material, further inquiries can be directed to the corresponding author.
